# Factors influencing treatment success of negative pressure wound therapy in patients with postoperative infections after Osteosynthetic fracture fixation

**DOI:** 10.1186/s12891-017-1607-0

**Published:** 2017-06-07

**Authors:** Kaywan Izadpanah, Stephanie Hansen, Julia Six-Merker, Peter Helwig, Norbert P Südkamp, Hagen Schmal

**Affiliations:** 1 Department of Orthopedic and Trauma Surgery, University Hospital of Freiburg, Freiburg, Germany; 2 Helmholtz Zentrum München, Munich, Germany; 3 Department of Orthopedics and Traumatology, Odense University Hospital and Department of Clinical Research, University of Southern Denmark, Sdr. Boulevard 29, 5000 Odense C, Denmark

**Keywords:** Infection, Osteosynthesis, Npwt, Vac, Clinical trial, Logistic regression models

## Abstract

**Background:**

Negative Pressure Wound Therapy (NPWT) is being increasingly used to treat postoperative infections after osteosynthetic fracture fixation. The aim of the present study was to analyze the influence of epidemiological and microbiological parameters on outcome.

**Methods:**

Infections following operative fracture fixation were registered in a comprehensive Critical Incidence Reporting System and subsequently analyzed retrospectively for characteristics of patients including comorbidity, bacteria, and clinical factors. The influence of the investigated parameters was analyzed using logistic regression models based on data from 106 patients.

**Results:**

Staged wound lavage in combination with NPWT allowed implant preservation in 44% and led to successful healing in 73% of patients. Fermentation characteristics, load and behavior after gram staining revealed no statistically significant correlation with either healing or implant preservation. Infecting bacteria were successfully isolated in 87% of patients. 20% of all infections were caused by bacterial combinations. We observed a change in the infecting bacterial species under therapy in 23%. Age, gender, metabolic diseases or comorbidities did not influence the probability of implant preservation or healing. The delayed manifestation of infection (>4 weeks) correlated with a higher risk for implant loss (OR 5.1 [95% CI 1.41–17.92]) as did the presence of bacterial mixture (OR 5.0 [95% CI 1.41–17.92]) and open soft-tissue damage ≥ grade 3 (OR 10.2 [CI 1.88–55.28]). Wounds were less likely to heal in conjunction with high CRP blood levels (>20 mg/l) at the time of discharge (OR 3.6 [95% CI 1.31–10.08]) or following a change of the infecting bacterial species under therapy (OR 3.2 [95% CI, 1.13–8.99]).

**Conclusions:**

These results indicate that the delayed manifestation of infection, high CRP blood levels at discharge, and alterations in the infecting bacterial species under therapy raise the risk of NPWT failure.

**Electronic supplementary material:**

The online version of this article (doi:10.1186/s12891-017-1607-0) contains supplementary material, which is available to authorized users.

## Background

Infections in trauma surgery are considered severe complications, because they often lead to persistent disability even in young patients, usually due to osteomyelitis or septic non-union. Several treatment approaches have been developed during recent decades, such as open wound treatment and the application of synthetic skin substitutes for wound closure as Grafix® (1) or NIKS-Based Bioengineered Skin Substitute Tissue (2). These procedures are all accompanied by the debridement of necrotic tissue to initiate regeneration of the surrounding soft tissue [1]. A modern treatment option for complex wounds (including traumatic injuries [2]) is to apply negative wound therapy delivered via the vacuum-assisted wound closure (VAC) therapy system [3]. VAC therapy has proven to be effective in the treatment of chronic wounds, including complex diabetic foot ulcerations [4, 5], resulting in a higher proportion of healed wounds, faster healing rates, and potentially fewer amputations compared with standard care. Other successful applications include the reliable fixation of mesh-grafts in reconstructive surgery [6], treatment of sternal osteomyelitis [7], and the closure of burn wounds [8]. Furthermore, NPWT appears to be a valuable adjunct for the treatment of traumatic wounds [9], reducing the number of necessary free muscle transfers, fasciocutaneous flaps or osteocutaneous flaps [10]. In addition to the treatment of traumatic wounds, NPWT therapy has become a basic procedure when treating postoperative infections after the open reduction and internal fixation of bone fractures.

Being located in different regions of the body, postoperative infections after osteosynthesis are multifaceted, and patients differ in their epidemiologic characteristics and comorbidities such as metabolic diseases. Moreover, the vast diversity in bacterial parameters (e.g. bacterial combination, identification of bacteria, changes in microbial strains during treatment) and soft-tissue traumas must be confronted during treatment.

This includes biofilm formation typically seen after infections with staphylococcus species. Bacteria adhere to the implants, form these extracellular polymers and, by this, are a major reason for bacterial persistence. This requires different treatment options including new generations of antimicrobial strategies (3). Furthermore, the diagnostic reliability of different sample drawing techniques and subsequent processing has been discussed extensively. Hereby, biopsies and culture of synovial fluid in blood culture vials has been shown to be more sensitive (90–92%) than intraoperative swab cultures (68–76%) (4).

The influence of these parameters on NPWT therapy’s effectiveness in postoperative infections following open reduction and internal fixation (ORIF) is unknown.

We therefore hypothesized that NPWT is a suitable supplement to support surgical treatment of postoperative wound infections. A further aim of this study was to analyze the impact of certain factors on the outcome after the treatment of infected osteosynthesis using NPWT therapy. To describe the outcome, we chose the parameters infection cure, implant survival, and the type of final wound closure. Primarily, we hypothesized that the efficacy of NPWT therapy in treatment of infections is influenced by parameters characterizing the infecting bacteria as gram staining or metabolism. Secondary, we hypothesized an influence of epidemiological parameters. Since the C-reactive protein is known as a reliable biomarker for monitoring acute inflammation [11] and postoperative healing success [12], this study further sought to address, whether there is a diagnostic threshold for the evaluation of healing in implant-associated infection.

## Methods

### Patients

All patients suffering an infection following open reduction and internal fixation who underwent NPWT therapy at our hospital were continuously registered using a Critical Incidence Reporting System (CIRS) over a period of 9 years (from 2001 to 2009) as described previously [13]. Our department used a noncommercial CIRS, which also provided a variety of other features, as a complication database. The first registration of a complication is done in the local documentation program of the Surgical Department (PROMetheus, Klinikrechenzentrum Freiburg, Germany). However, data were analyzed retrospectively. The recorded data are primarily documented by the treating surgeons, then checked for completeness and integrity by a database manager, and then randomly monitored during the quality control process twice a year (certification following DIN EN ISO 9001 by SGS-International Certification Services GmbH, Hamburg, Germany). Electronic documentation of surgical procedures included ICD and OPS codes and indications of postoperative complications. We requested a list of patients who had suffered postoperative infections after osteosynthesis and extracted those patients who had undergone NPWT/VAC therapy from the list. All those patients were included in the present study except those with pathologic fractures.

VAC procedures were evaluated regarding the infection’s successful healing (A), type of wound closure (B) and survival or preservation of the implant (C). These three categories were our outcome parameters. Two groups of wound closure were defined: group 1 included secondary wound closure and mesh-graft coverage, group 2 included all types of soft tissue coverage such as muscle flaps or full skin transplantations.

The data are presented as a retrospective consecutive case series based on reviewing a database (CIRS), containing continuously registered complications. Data was exported from the CIRS as it contains all VAC-treated infections at the authors’ institution. Over the documented years, the own infection rate pretty constantly ranged around 1.1%. Parameters such as patient characteristics (age, sex, co-morbidities, metabolic diseases), infection parameters (initial, maximum, and discharge CRP during hospitalization for revision surgery; time of infection, i.e. early onset [≤4 weeks] and late onset infection and extent of infection (soft tissue damage), bacterial parameters (gram stain, bacterial metabolism [fermentation], bacterial load, polymicrobial infection) and treatment parameters (intensive care unit stay in days, number of lavages until eradication [number of sponge changes; since all patients initially were treated by NPWT, 0 means that the sponge was applied once, but never changed], outcome of the implant [preservation or survival], re-infection) (see Table 1 for more information) were determined. All included cases were followed until either the infection has resolved or another definitive status had been documented such as amputation or chronic osteomyelitis. Overall, the follow-up of all patients was at least one year. The evaluation of the soft tissue damage was done according to the classification of Oestern and Tscherne, is a standard of the documentation, and was evaluated by the treating surgeon. The number of registered complications is a key process indicator for the certified quality management at our institution (ISO 9001®, KTQ®) and is required for yearly external audits; these data are therefore monitored regularly. This project was approved by our institutional ethical committee, and patients provided written informed consent for the use of their data for process control and scientific analysis.

**Table 1:** List of influencing parameters and outcome parameters defined in this study

**Influencing parameters** (Independent variables)	• **Patient parameters:** sex, age, co-morbidities, metabolic diseases (kidney diseases, liver diseases, diabetes mellitus) • **Infection parameters:** initial C-reactive protein (CRP), maximum CRP, discharge CRP (≤ 20 mg/l blood versus >20 mg/l)^a^, time of infection, extent of infection (superficial/deep), time point of infection (early [detected up to 4 weeks after surgery] or late onset [detected more than four weeks after surgery]), soft-tissue damage (according to the Tscherne and Oestern classification) • **Germal parameters:** bacterial mixture, identification of bacteria, change in microbial strain during treatment, gram stain, bacterial metabolism (aerobic/anaerobic), bacterial load (Group 1 – very high contamination, Group 2- numerous bacteria, Group 3-isolated bacteria, Group 4-bacteria detectable after enhancement) • **Treatment parameters:** Intensive care unit-stay, number of lavages until eradication, re-infection
**Outcome parameters **(Dependent variables)	• cure of infection • type of wound closure • preservation or survival of the implant

^a^CRP-discharge’ was calculated as CRP ≤20 mg/l blood versus >20 mg/l blood at the time of discharge or a change in the infecting bacterial species under therapy

### Treatment protocol

All patients included in the present study had undergone surgery. Staged lavage (every four to five days), local debridement and vacuum-assisted closure were performed with no exception. For Negative Pressure Wound Therapy (NPWT) exclusively products produced by KCI (KCI Medizinprodukte GmbH, Wiesbaden, Germany) were used. All wounds were treated by delivering of negative pressure at the wound site only without rinsing (no KCI V.A.C. Instill).

During each operative procedure, up to 5 superficial (subcutis) and deep smear tests (layer with osteosynthesis) were performed with pieces of debrided tissue for each patient. This was adapted and decided by the operating surgeon dependent on the local and the clinical situation and the verified bacterial infection status. The indication for each operative revision was based on local signs of infection such as pain, redness, pus, swelling, a foul odor, drainage or heat at the site combined with elevated serologic inflammatory parameters such as the leukocytic cell count and C-reactive protein (CRP). The indication for implant removal was given in case of persistent infection with clinical symptoms or insufficient soft tissue coverage. Antibiotic treatment was started in all cases with second-generation cephalosporin. If the bacteria were identifiable, the infection was treated with bacteria-specific antibiotics, and the pharmaceuticals were adapted accordingly. In cases of multi-resistant *Staphylococcus aureus* infections, vancomycin was selected for antibiosis. Patients presenting Pseudomonas aeruginosa infection underwent a specific antibiotic treatment according to tests of resistance. All other patients received continuous antibiotic treatment with second-generation cephalosporin. Indications for wound closure were negative smear tests from the previous operation, decreasing CRP blood levels and clinically good granulation of the wound base. If soft tissue conditions allowed adaption of the wound edges, a secondary suture was made. If not, split skin grafts were used for wound closure. Exposed bones, vessels, or nerves were covered via microsurgical techniques.

### Statistics

For the continuous variables, the mean with the standard deviation was obtained. Categorical sample characteristics were calculated as frequencies and percentages. Student’s t-test, the Kruskal-Wallis test, the Chi-Square test or Fisher’s Exact test was used to test differences in the patients’ distribution between the outcome parameters and the influencing parameters (see Tables 2 and 3 according to the statistical requirements). The normal distribution of continuous variables was checked by plotting a histogram and performing the Kolmogorov-Smirnov test, whereas the equality of variances was checked using Levene’s test Parameters with significant different distribution within the outcome parameters and sufficient sample size were incorporated initially into the logistic multiple Regression models. The main effect models were built using the backward elimination procedure. Using main effect regression models, the influence on the outcome parameter a successful restoration of the infection (Model A), the type of wound closure (Model B), and losing the implant before fracture healing had occurred (implant survival, Model C) were determined (Tables 4 and 5). The main effect Model A contained CRP discharge and change in microbial strain. Model B ‘type of wound closure’ could not be analysed due to no variable achieved the requirements. Model C contained bacterial mixture, time of infection and soft tissue damage. Results are presented as odds ratios (ORs) with 95% confidence intervals (CIs). Significance tests were two-tailed, and *P*-values lower than 0.05 were considered statistically significant. Parameters not documented within the CIRS, such as smoking, were not included. Analyses were performed using the statistical package SAS ® 9.2 (Cary, NC, USA).

**Table 2:** Summary of the collected data displaying distribution of the parameters investigated in the group with a cured infection and the group without.

Variable	Description	Cure of infection^a^						*p*-value^b^
		Yes			No			
		*n*	mean or %	SD	*n*	mean or %	SD	
Gender	Male	56	72.7		21	72.4		0.9743
	Female	21	27.3		8	27.6		
Age		77	53.2	18.9	29	55.8	17.9	0.5588
Stay at ICU	days	77	3.0	5.2	29	5.6	7.3	0.0790
Initial procedure	Open	1	1.3		2	6.9		0.1810
	Closed	76	98.7		27	93.1		
Tissue damage	Open Grade 0-2	11	14.3		8	27.6		0.0437
	Open Grade 3-4	7	9.1		6	20.7		
	Closed	59	76.6		15	51.8		
Identification of a	Yes	66	85.7		26	89.7		0.7535
microbial strain	No	11	14.3		3	10.3		
Presence of a bacterial mixture	Yes	12	15.6		9	31.0		0.2016
	No	54	70.1		17	58.6		
	No detectable germ	11	14.3		3	10.3		
Change in microbial strain	Yes	65	84.4		17	58.6		0.0047
	No	12	15.6		12	41.4		
Gram stain	Positive	57	74.0		17	58.6		0.0746
	Negative	9	11.7		9	31.0		
	No detectable germ	11	14.3		3	10.4		
Bacterial metabolism	Aerobic	3	3.8		4	13.8		0.0309
	Anaerobic	1	1.3		2	6.9		
	Facultative anaerobic	60	77.9		16	55.2		
	Facultative aerobic	2	2.6		3	10.3		
	No detectable germ	11	14.3		4	13.8		
Bacterial load	Plentiful	18	23.4		9	31.0		0.9095
	Numerous	25	32.5		7	24.1		
	Sporadic	20	26.0		8	27.6		
	Enrichment	3	3.9		1	3.4		
	No detectable germ	11	14.3		4	13.8		
Number of lavages until eradication	sponge changes 0	33	42.9		18	62.1		0.0431
	sponge changes 1	20	26.0		6	20.7		
	sponge changes 2	14	18.2		0	0.0		
	sponge changes ≥3 times	8	10.4		3	10.3		
	Never be eradicated	2	2.6		2	6.9		
CRP Initial	[mg/l]	72	78.2	92.1	25	69.6	78.4	0.8112
CRP Maximum	[mg/l]	76	128.8	101.7	25	137.5	94.6	0.4547
CRP Discharge	[mg/l]	75	16.3	23.6	26	46.1	52.1	0.0077
CRP Discharge	≤20 mg/l	58	77.3		12	46.2		0.0036
	>20 mg/l	17	22.7		14	53.8		
Time point of infection	Early	49	63.3		16	57.1		0.5446
	Late	28	36.7		12	42.9		
Extent of infection	Superficial	2	2.6		1	3.4		1.000
	Deep	75	97.4		28	96.6		
Metabolic disease^c^	Yes	12	15.6		7	24.1		0.3061
	No	65	84.4		22	75.9		
Re-infection	Yes	23	29.9		14	48.3		0.0764
	No	54	70.1		15	51.7		
Implantat preservation	After fracture consolidation	40	53.3		7	24.1		0.0073
	Before fracture consolidation	35	46.6		22	75.9		

SD = standard deviation, an total = 106, b in all analysis two-tailed tests with a significance level of 0.05 were used, ^c^ metabolic diseases = kidney diseases, liver diseases and diabetes mellitus

**Table 3:** Summary of the collected data displaying distribution of the parameters investigated in the group with implant survival/preservation and the group in whom the implant was removed

Variable	Description	Survival of the implant until bony consolidation^a^						*p*-value^b^
		Yes			No			
		*n*	Mean or %	SD	*n*	Mean or %	SD	
Gender	Male	38	80.9		37	64.9		0.0712
	Female	9	19.1		20	35.1		
Age		47	55.5	18.5	57	52.1	18.9	0.3006
Stay at ICU	days	47	4.5	6.5	57	3.1	5.5	0.4337
Initial procedure	Open	45	95.7		1	1.8		0.5881
	Closed	2	4.3		56	98.2		
Tissue damage	Open Grade 0-2	7	14.9		11	19.3		0.042
	Open Grade 3-4	2	4.3		11	19.3		
	Closed	38	80.9		35	61.4		
Identification of a	Yes	43	91.5		48	84.2		0.2640
microbial strain	No	4	8.5		9	15.8		
Presence of a bacterial mixture	Yes	5	10.6		15	26.3		0.0413
	No	38	80.8		33	57.9		
	No detectable germ	4	8.5		9	15.8		
Change in microbial strain during treatment	Yes	40	85.1		40	70.2		0.0721
	No	7	14.9		17	29.8		
Gram Stain	Positive	34	72.3		39	68.4		0.8615
	Negative	8	17.0		10	17.5		
	None	5	10.6		8	14.0		
Bacterial metabolism	Aerobic	3	6.4		4	7.0		0.2172
	Anaerobic	1	2.1		2	3.5		
	Facultative anaerobic	38	80.9		37	64.9		
	Facultative aerobic	0	0.0		5	8.8		
	No detectable germ	5	10.6		9	15.8		
Bacterial load	Plentiful	10	21.3		17	29.8		0.7030
	Numerous	17	36.2		15	26.3		
	Sporadic	13	27.7		14	24.6		
	Enrichment	2	4.3		2	3.5		
	No detectable germ	5	10.6		9	15.8		
Number of lavages until eradication	sponge changes 0	23	48.9		27	47.4		0.6821
	sponge changes 1	13	27.7		13	22.8		
	sponge changes 2	4	8.5		9	15.8		
	sponge changes ≥3 times	7	14.9		4	7.0		
	Never be eradicated	0	0.0		4	7.0		
CRP Initial	[mg/l]	45	81.5	94.1	50	72.6	85.1	0.7201
CRP Maximum	[mg/l]	47	147.6	102.6	52	115.2	95.2	0.2187
CRP Discharge	[mg/l]	46	23.2	39.2	53	24.9	32.9	0.6668
CRP Discharge	≤20 mg/l	34	73.9		35	66.0		0.5113
	>20 mg/l	12	26.1		18	34.0		
Time Point of Infection	Early	36	76.6		27	48.2		0.0032
	Late	11	23.4		29	51.8		
Extent of Infection	Superficial	0	0.0		2	3.5		0.4998
	Deep	47	100.0		55	96.5		
Metabolic disease^c^	Yes	8	17.0		9	15.8		0.8657
	No	39	82.0		48	84.2		
Re-infection	Yes	14	29.8		23	40.4		0.3951
	No	33	70.2		34	59.6		
Curing the Infection	Yes	40	85.1		35	61.4		0.0086
	No	7	14.9		22	38.6		

SD = standard deviation, ^a^n total = 106, missing data on implant survival until bony consolidation n = 2, ^b^ in all analysis two-tailed tests with a significance level of 0.05 were used, ^c^ metabolic diseases = kidney diseases, liver diseases and diabetes mellitus

**Table 4:** Logistic regression models on the outcome parameter “infection cured”

Cure of Infection		Multiple logistic regression model^a^		
Influencing Parameters	vs.	OR	[95% CI]	*p*
CRP – discharge ≤ 20 mg/l	> 20 mg/l	3.6	[1.31-10.08]	0.0134
Change of microbial strain Yes	No	3.2	[1.13-8.99]	0.0289

All p-values presented are two-tailed, and p-values lower than 0.05 were considered statistically significant, 95% confidence level (CI)

^a^outcome: infection cured (yes/no), independent variables: CRP – discharge (≤20 mg/l vs. >20mg/l), change in microbial strain (yes/no), and survival of the implant until bony consolidation (yes/no)

**Table 5:** Logistic regression models on the outcome parameter “implant survival”

Survival of the implant	Multiple logistic regression model^a^			
Influencing Parameter	vs.	OR	[95% CI]	*p*
Bacterial mixture Yes	No	5.0	[1.41–17.92]	0.0126
No detectable germ	No	3.4	[0.82–14.11]	0.0932
Time point of infection late	acute	5.1	[1.93–13.41]	0.0010
Soft tissue damage				
Open grade 0–2	Closed	2.6	[0.76–8.78]	0.1262
Open grade 3–4	Closed	10.2	[1.88–55.28]	0.0072

All *p*-values presented are two-tailed, and *p*-values lower than 0.05 were considered statistically significant, 95% confidence level (CI)

^a^outcome: implant survival until bony consolidation (yes/no), independent variables: bacterial mixture (dummy coding (yes/no (reference category)/no detectable germ), time of infection (postoperative/late onset), and soft tissue damage (dummy coding (open grade 0–2/open grade 3–4/closed (reference category)

## Results

### General

One hundred and six patients were enrolled in the present study, most of whom were male (77 males, 29 females). Average age was 54 (SD 19) years. Twenty-two of these patients suffered from metabolic diseases such as diabetes mellitus (7/6.7%), hyper- or hypothyroidism (1/0.9%), renal insufficiency (3/2.8%) or hepatic insufficiency (9/8.5%). Two patients suffered from chronic myeloid leukemia, one from laryngeal carcinoma and two from chronic occlusive disease (Grade 2). Patients suffering chronic myeloid leukemia received low dose prednisolone.

In 53 patients a grade 1 closed fracture, in 10 patients a grade 3 closed fracture, in four patients a grade 1 open fracture, in 22 patients a grade 2 open fracture and in 17 patients a grade 3 open fracture was documented. 4 Patients suffered from occlusive arterial disease, in 5 patients an alcohol addiction was documented. In 9 patients a BMI > 25 was recorded.

The distribution of fractures was as follows: femur (22/21.0%), lower leg (42/40.0%), foot (12/11.0%), clavicle (1/0.9%), upper arm (7/6.6%), forearm (5/4.7%), pelvis (6/5.6%), and spine (11/10.4%).

Seventy infections (66.0%) occurred after plate osteosynthesis; 20 (18.9%) occurred after osteosynthesis involving only pins, wires or screws; 11 (10.4%) occurred after implantation of an internal fixator; and 5 (4.7%) occurred after intramedullary nailing.

**Fig. 1:**
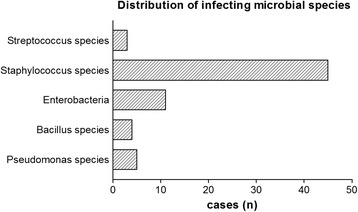
The figure provides an overview about the infecting microbial species

In 87% of all treated patients, bacteria were isolated during primary lavage; a polymicrobial infection was present in 20% of the cases. *Staphylococcus aureus* (42/39.6%) and *Staphylococcus epidermidis* (12/11.3%) were identified as the predominant cause of infection. Furthermore, Bacillus species (5/4.7%), *Escherichia coli* (2/1.8%), *Enterobacter aerogenes* (1/0.9%), *Enterobacter cloacae* (4/3.6%), Enterococcus faecalis (4/3.6%), Enterococcus faecium (2/1.8%), Methicillin Resistant *Staphylococcus aureus* (6/5.6%), Peptostreptococcus species (2/1.8%), Proteus mirabilis (1/0.9%), Pseudomonas aeruginosa (7/6.3%), Staphylococcus auricularis (1/0.9%), Staphylococcus capitis (1/0.9%), Staphylococcus haemolyticus (2/1.8%), Staphylococcus lugdunensis (1/0.9%), Stenotrophomonas maltophilia (2/1.8%), *Streptococcus agalactiae* (2/1.8%), and Streptococcus equisimilis (1/0.9%) were isolated. An overview provides Fig. 1.

Gram staining was positive in 80% of the samples and negative in 20%. 7.3% of the isolated bacteria were aerobic germs, 3.1% were anaerobic germs, 79.2% were facultative anaerobic germs, and 5.2% were facultative aerobic germs. The microbial strains changed under therapy in 23% of all cases.

In summary, infection healing was achieved in 73% of all cases in the present study. A locally controlled osteomyelitis persisted in 16% of all cases, and 4% underwent an amputation. 44% of all implants survived at least until bony consolidation, and only secondary suturing or mesh grafting had to be done for wound closure in 78% of patients.

### Curing the infection

There were no significant differences in gender, age, intensive care unit-treatment (ICU-treatment), identification of a microbial strain, bacterial load, type of bacteria, presence of a bacterial mix, CRP at admission to the hospital, or maximum CRP measured during the stay in patients whose infection was cured compared to patients who developed a chronic infection (Table 2). We detected significant differences regarding the presence of soft tissue damage, a change in microbial strain during treatment, bacterial metabolism, and the distribution of CRP at the time of discharge (Table 2).

Due to small case numbers in some subgroups, we conducted no statistical testing for the influence of the infection’s location on its cure (data not shown).

In the main effect multiple logistic regression model, the ORs for patients with a CRP at discharge ≤20 mg/l and change in microbial strain were 3.6 [95% CI 1.31–10.08] and 3.2 [1.13–8.99], respectively.

To evaluate the influence of comorbidity, the Charlson comorbidity index score (CCoMI) was assessed as recently described (5). For reasons of statistical practicability, all patients with an CCoMI of 0 (78%) were compared to all patients with an CCoMI > 0 (22%). Although the infection could be successfully cured in the first group in 82% and in the second group only in 67%, there was no statistically significant difference. Furthermore, the analysis of the subgroups with oncological, cardiovascular, liver or renal comorbidity, polytrauma or substance abuse failed to show statistical differences, but resulted only in small sized subgroups.

### Types of wound closure

We identified no significant association between any of the parameters when comparing patients who underwent a minor reconstructive procedure such as secondary wound closure or mesh graft coverage with those undergoing plastic reconstructive treatment such as muscle flap coverage.

### Preservation (survival) of the implant

When comparing patients whose implants survived with those whose implants were removed, we observed no significant difference in the distribution of gender, age, treatment at an intensive care unit, change in microbial strain during therapy, identification of a specific bacteria, bacterial load, CRP at admission to the hospital, maximum CRP measured during the stay, CRP at the time of discharge, metabolic diseases, or extent of soft tissue damage. Implant survival was statistically significantly influenced by the presence of a bacterial mixture (*p < 0.05*) and by the timing of the infection (primary vs. secondary) (*p < 0.01*). When the infection had been cured, the implant was preserved more often (*p < 0.01*) (Table 3). There were no identified maximal numbers of revisions; despite more than 3 revisions in some cases the implant could be preserved.

The main effect multiple logistic regression models revealed that a late-onset infection yielded a 5.1-fold higher odds ratio [95% CI 1.41–17.92] of losing the implant before bony consolidation compared with early-onset postoperative infections. We also noted further associations between the presence of a bacterial mixture and secondary wound infections (OR 5.0 [95% CI 1.41–17.92] and OR 10.2 [95% CI 1.88–55.28] for losing the implant before bony consolidation (see Table 5).

## Discussion

Key findings of the present study are that the patient’s age, gender or co-morbidities do not significantly impair the likelihood of a successfully cured infection, implant survival until bony consolidation, or the type of wound closure during NPWT therapy of infected osteosynthesis. Moreover, NPWT therapy proved to be equally effective in gram-positive and gram-negative aerobic and anaerobic bacteria with regards to our outcome parameters. However, the primary presence of a bacterial mixture or changes in microbial strains during therapy increased the risk that bony consolidation was only feasible after implant removal. The C-reactive protein serum level is a good parameter for monitoring the success of treatment. Indeed, a critical concentration of less than 20 mg/l (norm ≤5 mg/l) appears to indicate successful treatment of infection.

Implant-associated infections after the treatment of closed fractures in the skeletal system occur in 0.2–9.0% [14] of cases, a complication that might be reduced significantly by regular perioperative application of single-injection antibiotics [15]. Variables such as tissue viability and damage, the presence of infection, exposed osteosynthetic material, implant failure, fracture location and patient-related factors contribute to the lack of general consensus regarding the management of these defects [1], [16]. Most authors recommend an initial radical debridement followed by early implant removal, permanent drainage [14], the application of local antibiotics [17], vacuum-assisted closure (VAC) of the wound, or a combination of these approaches. There is evidence that adequate early wound coverage, preferably with vital tissue, results in fewer infections [18]. We carried out this study because the efficacy of NPWT therapy had not been examined in conjunction with the treatment of fracture implants. Our data show that administering NPWT therapy for implant-associated infections after osteosynthetic stabilization facilitates implant preservation in 44% of cases and healing with secondary wound closure in 73% of cases, which confirms our initial hypothesis and specifies treatment expectations. The predominant bacterial strains isolated in the present study, such as *Staphylococcus aureus* and *Staphylococcus epidermidis,* are consistent with other findings regarding implant-associated infections [19]. NPWT therapy was known to be effective in the treatment of surgical infection sites [20], but it was unclear whether the efficacy of NPWT treatment depended on specific bacterial characteristics such as gram staining or aerobic/anaerobic metabolism, until now. The potential formation of biofilms has been identified as a risk factor for persistent and recurrent infections [21], a phenomenon associated with specific microbial strains. Our data do not permit us to conclude that those features of bacterial biology influence NPWT therapy’s effectiveness in treating infected implants. Neither the initial bacterial load nor initial presence of several bacterial strains correlated with an unfavorable outcome in our patients. Not until isolated bacterial strains had changed during treatment did the risk of implant removal rise significantly. This result appears to concur with findings from a prospective randomized trial [22]. That working group demonstrated a positive NPWT-therapy effect on wound healing that was associated with a significant reduction in the wound surface area, although the effect could not be attributed to a quantitative reduction in the bacterial load [22]. Some researchers have even reported increased bacterial colonization during NPWT therapy [23]. Despite this potentiality, the beneficial effects of this treatment modality on wound healing outnumber the negative effects [23]. The present data indicate only indirectly that biofilm formation is a key, because secondary or “low grade” infections with delayed diagnosis correlated with significantly lower probability for successful treatment. However, since we did not analyze biofilm formation directly, we can only hypothesize that this is a factor for the higher failure rate in delayed onset infection. The monitoring of infections with specific serum markers is of clinical importance [11] because reliable biomarkers are essential for decision making in therapy. CRP has been validated as a sensitive control parameter for diagnosing infections in arthroplasty [24] and for monitoring healing processes following joint surgery [12]. Our data confirm those findings, although only the CRP at discharge, but not initial or maximum CRP values did correspond to the final outcome. In fact, CRP levels at the day of discharge >20 mg/l (normal ≤5 mg/l) did associate strongly with the failure of infection clearance. Therefore, in patients whose implants are infected, this parameter should return to normal levels. Age > 60 years, smoking, diabetes, previous surgical infections, increased body mass index, and alcohol abuse are known to be significant preoperative risk factors [25] for perioperative infections. It had been unclear, whether these parameters also influence the efficacy of NPWT therapy. Analysis of our data showed that age, gender, treatment in an intensive care unit, or co-morbidities such as metabolic diseases were not associated with a higher risk for an unfavorable outcome. Furthermore, the data from the present study revealed that the delayed onset of an infection and persistent high CRP blood levels during therapy correlate negatively with the healing of a postoperative infection. These effects do not appear to be dependent on the thickness of the surrounding soft tissue layers or on the fracture site.

A limitation of the study is that the CIRS was not primarily designed to identify and evaluate the outcome parameters we examined (infection cure, types of wound closure, and implant survival). Furthermore, we consolidated the infected implants in different locations to attain reasonable case numbers to analyze different factors. The analysis of the comorbidity resulted in the same problem, again the case numbers were too small to reach statistical significances regarding diseases known to have more soft tissue complications. This issue is common when assessing general treatment strategies in the field of orthopedic and trauma surgery, because fracture sites are so diverse and the degree of damage can also differ. However, these limitations may be balanced in part by applying the appropriate statistical model for analysis. In the present study, a multiple regression model was established. This enabled our results to be adjusted for specific parameters. The inherent bias of these parameters could thus be minimized and the real strength of the correlations identified. However, the small numbers of patients in this group may have yielded misleading effects during statistical evaluation. Moreover, as no control group was involved, we cannot claim that the validity of our findings is limited to the administration of NPWT therapy for infected osteosynthesis or that they can be generalized.

As consequence for clinical decision making, a re-infection rate of almost 30% needs to be seen critically. Instead of retaining the implant, it might be removed accompanied by external fixation and local debridement. Only in some selected cases it might be worthwhile to keep the implant, especially in early onset infections with early diagnosis.

## Conclusion

In conclusion, the patient’s age, sex and comorbidities do not significantly influence the success of NPWT therapy. The delayed onset of infection, changes in bacterial strains during treatment, and elevated CRP levels at discharge do increase the risk for treatment failure.

## Additional file

Additional file 1: “anonymized data set VAC” (XLS 121 kb)

## Abbreviations

CCoMI: Charlson comorbidity index score
CI: confidence interval
CIRC: Critical Incidence Reporting System
CRP: C-reactive protein
ICD: International classification of diseases
ICU: intensive care unit
OPS: Operationen- und Prozedurenschlüssel (code for operations and procedures)
OR: Odds ratio
VAC: Vacuum-assisted wound closure
